# Temporal Instability of Factors Affecting Injury Severity in Helmet-Wearing and Non-Helmet-Wearing Motorcycle Crashes: A Random Parameter Approach with Heterogeneity in Means and Variances

**DOI:** 10.3390/ijerph191710526

**Published:** 2022-08-24

**Authors:** Muhammad Ijaz, Lan Liu, Yahya Almarhabi, Arshad Jamal, Sheikh Muhammad Usman, Muhammad Zahid

**Affiliations:** 1 School of Transportation and Logistics, Southwest Jiaotong University, Chengdu 610031, China; 2 Center of Excellence in Trauma and Accidents, King Abdulaziz University, Jeddah 21589, Saudi Arabia; 3 Department of Surgery, Faculty of Medicine, King Abdulaziz University, Jeddah 21589, Saudi Arabia; 4 Transportation and Traffic Engineering Department, College of Engineering, Imam Abdulrahman Bin Faisal University, P.O. Box 1982, Dammam 31451, Saudi Arabia; 5 Department of Civil Engineering, CECOS University of I.T. & Emerging Sciences, Peshawar 25000, Pakistan; 6 College of Metropolitan Transportation, Beijing University of Technology, Beijing 100124, China

**Keywords:** helmet-wearing, non-helmet-wearing, motorcyclists, random parameters logit model, temporal instability, Rawalpindi

## Abstract

Not wearing a helmet, not properly strapping the helmet on, or wearing a substandard helmet increases the risk of fatalities and injuries in motorcycle crashes. This research examines the differences in motorcycle crash injury severity considering crashes involving the compliance with and defiance of helmet use by motorcycle riders and highlights the temporal variation in their impact. Three-year (2017–2019) motorcycle crash data were collected from RESCUE 1122, a provincial emergency response service for Rawalpindi, Pakistan. The available crash data include crash-specific information, vehicle, driver, spatial and temporal characteristics, roadway features, and traffic volume, which influence the motorcyclist’s injury severity. A random parameters logit model with heterogeneity in means and variances was evaluated to predict critical contributory factors in helmet-wearing and non-helmet-wearing motorcyclist crashes. Model estimates suggest significant variations in the impact of explanatory variables on motorcyclists’ injury severity in the case of compliance with and defiance of helmet use. For helmet-wearing motorcyclists, key factors significantly associated with increasingly severe injury and fatal injuries include young riders (below 20 years of age), female pillion riders, collisions with another motorcycle, large trucks, passenger car, drivers aged 50 years and above, and drivers being distracted while driving. In contrast, for non-helmet-wearing motorcyclists, the significant factors responsible for severe injuries and fatalities were distracted driving, the collision of two motorcycles, crashes at U-turns, weekday crashes, and drivers above 50 years of age. The impact of parameters that predict motorcyclist injury severity was found to vary dramatically over time, exhibiting statistically significant temporal instability. The results of this study can serve as potential motorcycle safety guidelines for all relevant stakeholders to improve the state of motorcycle safety in the country.

## 1. Introduction

Globally, traffic crashes cause over 1.35 million deaths every year [1,2]. Estimates suggest that by 2030, traffic crashes will become the fifth largest cause of human fatalities worldwide [3,4,5]. Drivers and riders of motorized three-wheeler and two-wheeler bikes comprise 28% of these fatalities. Motorcyclists, being less protected, are at a high risk of severe injuries compared to other road users. It has been reported that motorcycle riders are at a 34-fold higher risk of fatal crashes than other car types [6]. Moreover, head injuries are the primary source of trauma and casualties for these riders [7]. Motorcycle injuries account for a significant proportion of all traffic casualties in Pakistan, and bike riders are most likely to experience severe injury among vulnerable road users. In recent years, several enforcement policies such as helmet use among drivers, child restraint law, and the provision of dedicated lanes have been proposed at the national level. Helmet usage among motorcyclists has been reported to prevent or significantly mitigate the chances of traumatic head and brain injuries [8]. A systematic review published in 2008 showed that riders who wore helmets had a 69% and 42% lower risk of head injuries and deaths, respectively.

Non-helmet is a key risk factor in fatal motorcycle crashes. These injuries can be avoided by adopting rules that enforce road safety measures, including helmet use. In a global investigation, researchers discovered that the non-use of a helmet was the most important factor influencing a motorcyclist’s fatality rate in an RTI and that helmets decrease the likelihood of death in a crash [9]. Furthermore, a study in Taiwan discovered that head and brain injuries among motorcycle riders without helmets were four and ten times more frequent, respectively [10]. Additionally, improper helmet attachment and the usage of substandard helmets are two further secondary concerns that are extremely significant in LMICs [9]. Additionally, head injuries appear to be more common and severe for riders who use a non-standard helmet than for those who wear a standard helmet [11]. Borrowed and poorly fitted helmets are commonly reported in many developing countries, and more than one-third of motorcyclists showed inappropriate helmet use, such as wearing it on the back of the head and having a loose chin strap. The study’s findings showed that utilizing non-standard helmets (such as sun, bamboo, aging plastic, cheaper helmets, and so on might limit the predicted benefits of helmet usage initiatives [12,13].

Driver-related factors are reported to be the leading contributors to traffic accidents worldwide for all modes of transportation [14,15,16,17,18,19,20,21,22]. The motorbike riders’ helmet usage and its effect on safety have been the subject of several previous studies. A study conducted by [23] investigated the effect of expanding helmet use on diminishing severe injuries and fatalities experienced by bike riders in Thailand. The analysis was based on two years before (1994 to 1995) and after (1996 to 1997) motorcyclists’ crash data containing 12,002 instances. Study results revealed that helmet use reduced the associated deaths by 20.8% and head injuries by over 41%. The study also reported more compliance with helmet use during night than daylight conditions. Another study [24] utilized repeated cross-section crash data of individual motorcyclists and attempted to examine the relationship between mandatory helmet legislation and the resulting fatalities. It was observed that motorcyclists following obligatory helmet laws were approximately 30% less liable to receive traffic violation tickets for risky and aggressive driving. In addition, adopting universal helmet laws led to an average fatality reduction of 20.5%. Likewise, non-incapacitating and incapacitating motorcycle injuries were lowered by 13.5% and 5.1%, respectively. A recent study conducted by [25] analyzed the injury patterns and severity of motorcyclists exhibiting compliance with and defiance of helmet use in a rural state of Maine in the U.S. Driver sociodemographic data, injury, and patient result were additionally considered. Patients were delineated as not wearing a helmet and unknown helmet use. Statistical analysis of the data was conducted through one-way ANOVA or Pearson’s χ^2^, or Student’s *t*-test, using a *p*-value = 0.05 for identifying the critical factors. Study results suggested that helmet users had fewer face and head injuries, fewer cervical spine injuries, and a lower severity of crash injuries. Similarly, helmet-wearing riders had, altogether, a reduced ICU stay and fewer mechanical ventilation post-crash events.

A recent study [26] employed ANOVA to examine the severe head injuries in motorcycle crashes involving compliance with and defiance of helmet use. Out of 906 patients surveyed (wearing helmet = 701 and not wearing helmet = 205), the mean severity was 9.3 ± 6.4. The prevailing injury types were rib, concussion, clavicle, cervical spine, and facial fractures. Generally, the review results showed that helmet use is related to fewer chances of severe head injuries and facial or skull fractures [27], and also examined the incidence and patterns of maxillofacial injuries in motorcycle crash patients with and without helmet usage. Statistical analysis of 717 cases revealed that non-helmeted riders had higher orbital fractures, maxillary malar, abrasion, and soft tissue contusions. The study also reported that helmet use not only protects against head injuries but is also effective in reducing midfacial fractures.

In the study [28], the researchers studied different elements influencing helmet usage behavior and developed a predictive model for estimating helmet usage behavior in a developing country. Data were collected in time frames, i.e., before and after the mandatory helmet law, at ten random locations in Mumbai, India. Kendall’s tau, Pearson’s R, Spearman’s rho correlation, and analysis of variance tests were performed to identify each predictor variable’s impact on motorcycle riders’ helmet usage behavior. To estimate the helmet usage behavior of riders, binary logistic regression models were developed. The study concluded that helmet usage increased by over 20% following the mandatory helmet law, from 62.81% to 83.51%. The study results also suggested that the survey interval, land use pattern, type of helmet, number of occupants, driver age, and gender highly influence the rider’s helmet usage behavior. Female riders were found to wear helmets more frequently than their male counterparts. The rate of non-helmet usage was exceptionally high during early morning peak periods due to the absence of traffic police personnel. A study conducted by O’Connor in 2005, investigated the role of the helmet and helmet type on the injury examples of riders [29]. The outcomes uncovered that there was no massive distinction in injury examples of riders regardless of helmet use. Similarly, helmet usage was higher among riders with children and women as pillion passengers.

A similar study [30] reported that the need for or inappropriate use of helmets among bike riders is associated with a higher probability of fatal accidents. A recent study [31] conducted a ureteroscopy study across nine low- and middle-income countries to evaluate the safety performance of motorcycle helmets. The study findings suggested that using non-standard helmets might restrict the expected advantages of helmet usage programs. In another study [32], the researchers compared the injury severity outcome of helmet-wearing and non-helmet-wearing motorcycle riders in north-western Tanzania. A total of 654 cases of victims involved in motorcycle crashes were examined. Helmet usage was reported in 47.7% of patients, with young riders not wearing a helmet outnumbering the helmet users in other age groups. It was also revealed that the non-usage of a helmet was significantly associated with severe abnormal head injuries, severe trauma, admission to emergency ICUs, and worse traumatic brain injuries.

In a recent study [33], authors performed a retroscopic review of factors associated with helmet usage in Southeast Asian nations, focusing on adopted interventions. The effectiveness of helmet enforcement laws on a qualitative scale (between 0 = ineffective to 10 = highly effective) showed that Malaysia had the lowest (5) while Brunei Darussalam (10) had the best ratings. The average ratings for all the countries in the region were 7.2, implying that helmet usage is only partially practical. On the other hand, helmet usage was linked with a reduced mortality rate and shorter periods of hospitalization. [34] examined the motorcycle helmet trends and usage before and after the Florida helmet law change in 2000. The law change provided permission to not use a helmet for riders aged 21 or above and carry threshold insurance (worth USD 10,000) to cover medical costs incurred due to crash occurrence. Findings showed that the helmet use rate declined by nearly 50% in 2002 following the law change. However, crash and injury rates per motorcycle and motorcycle vehicle miles traveled (VMT) continued to decline following the law change except for fatal crash rates.

Another study [35] studied the effect of required helmet use on the frequency and severity of motorcycle crashes in Uruguay. The study utilized synthetic control and differences in methods and found that helmet use was related to an approximately 40% reduction in severe and fatal crashes. The change translated to an increase in minor injuries. In another study [36], the authors examined the patterns of head injuries sustained by helmet-wearing and non-helmet-wearing bike riders. The results indicated that proper helmets could effectively protect against extra and intracranial head injuries. Another study [37] evaluated the effect of motorcyclists’ helmet and drug usage on riders’ injury severity. Study results showed that bike riders not wearing helmets had a worse crash outcomes than their counterparts, irrespective of alcohol or drugs use. [38] also studied the effects of helmet use on rider injury severity and motorcycle crash trauma. The study results showed injuries pertaining to three specific AIS body parts, i.e., head, chest, and abdomen, had significantly different severities between helmet-wearing and non-helmet-wearing users. The head and abdomen of non-helmet-wearing motorcyclists were more severely injured. The use of helmets was observed to decrease head injuries significantly. Another study [39] reported that the helmet usage rate among children was less than among adults in Luang Prabang, Laos. The study also highlighted the prevailing and perceived reasons for non-compliance to helmet usage among the surveyed populations. The study [40] also showed that helmet use effectively reduces head, face, and brain injuries among motorcyclists.

According to a local viewpoint, a critical bibliographic investigation of the current study has shown that crash injury severity involving helmet-wearing and non-helmet-wearing motorcyclists is seldom addressed. This study aims to involve the discoveries of the previous exploration as a foundation for obtaining how helmet and non-helmet-wearing motorcyclists affect injury severity outcomes. Table 1 summarizes previous researchers’ approaches to estimating injury severity associated with helmet and non-helmet-wearing motorcyclists. Given this, the study’s primary objective is to explore the temporal instability and non-transferability of injury severities among helmet and non-helmet-wearing motorcyclists. We are especially keen on seeing how the impact of variables affecting injury severities results changes in helmet- and non-helmet-wearing motorcycle’ crashes over time. A random parameter logit model with heterogeneity in means and variance was evaluated to assess and define primary risk factors in helmet-wearing and non-helmet-wearing motorcycle crashes for the current study area is the secondary objective of the study. The findings could be of great value to policymakers and safety practitioners in promoting motorcycle safety.

## 2. Data Description

**Figure 1:**
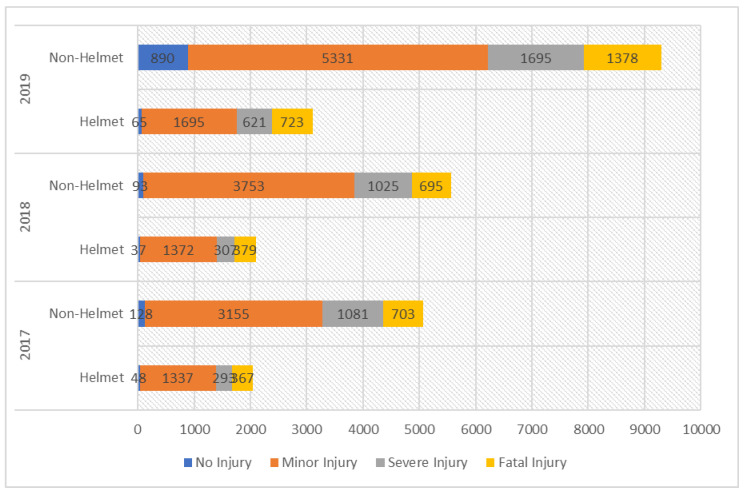
Motorcyclist’s injury severity for helmet and non-helmet motorcycle crashes over the years: 2017–2019.

This study uses three-year crash data from the road traffic accident database of RESCUE 1122, the leading emergency service in Pakistan, on motorcycle crashes involving helmet-wearing and non-helmet-wearing riders. This study focuses on motorcycle crashes in Rawalpindi city for three years, 2017, 2018, and 2019. The final dataset includes 24,237 crash reports with information on victims’ demographic characteristics, such as age and gender of the rider, crash point, crash date and time, cars involved in the crash, rider details, and motorcycle details on the spot. The study also collected additional information, such as weather information at the crash time and roadway geometric features from Rawalpindi Development Authority and Pakistan Meteorological Department respectively. Injury severity levels are categorized into four ordinal scales: no injury, minor injury, severe injury, and fatal injury. Figure 1 describes the injury severity distribution for helmet-wearing and non-helmet-wearing motorcyclists across the three-year study period (2017–2019). This figure shows a noticeable difference in helmet-wearing vs. non-helmet-wearing motorcyclists’ injury severities, with non-helmet-wearing motorcyclists being more associated with crashes resulting in serious injuries. Table 2 gives descriptive measurements of statistically significant logical factors in at least one of the six models assessed (models for helmet-wearing and non-helmet-wearing motorcyclists’ injury severity for each of the three years analyzed).

## 3. Methodology

### 3.1. Modeling Approach

Numerous researchers have focused on random parameter approaches for accounting for the unobserved heterogeneity [52,53,54,55,56,57,58,59]. A random parameter logit model with heterogeneity in means and variances was used in this study to estimate the injury severity of helmet-wearing and non-helmet-wearing motorcyclists. The modeling approach starts with the severity function that defines individual crash *n* as the injury severity *i*. Motorcyclist crash injury severity is divided into four categories: no injury, minor injury, severe injury, and fatal injury.

(1) $$M_{in}=\beta_{i}X_{in}+\varepsilon_{in}$$

*M_in_* is motorcyclist injury seriousness work assessing seriousness for level I (no injury, minor injury, extreme injury, and lethal injury) for crash *n*, Xin is a vector of logical variables, *β_i_* is a vector of respectable parameters, and *ε_in_* is a term for stochastic error. Unnoticed heterogeneity in means and fluctuations of arbitrary boundaries are provided for by enabling *β_i_* to be a vector of outstanding limits that shifts across crash perceptions distinguished by [58,60,61,62,63,64,65] as follows

(2) $$\beta_{in}=\beta \;+\;\Theta_{in}Z_{in}\;+\;\sigma_{in}EXP(\omega_{in}W_{in})v_{in}$$

where *β* is the average value of parameter across all crashes, *Z_in_* is a vector of estimable boundaries that keeps the heterogeneity in the mean, *Θ_in_* is a relating vector of estimable boundaries. *W_in_* is a vector of estimable boundaries that records heterogeneity in standard deviation *σ* in with relating vector *ω_in_*, and *υ_in_* represents the disturbance term. Expecting the blunder term εin to be summed up with a wide range of values, the subsequent standard multinomial logit model likelihood that empowers variables to shift across perceptions is characterized as [66]

(3) $$P_{n}\left(i\right)=\frac{EXP\left[\beta_{i}X_{in}\right]}{\sum_{\forall I}EXP[\beta_{i}X_{in\;}]}$$

where *P_n_*(*i*) is the probability of motorcyclist crash n leading to severity outcome *i*, whereas *i* is a set of all possible injury severity outcomes. To permit at least one boundary gauge in the vector *β_i_* to shift across individual accident perceptions, the above equation is modified as [62,64,65]

(4) $$P_{n}\left(i\right)=\int \frac{EXP\left(\beta_{i}X_{in}\right)}{\sum_{\forall I}EXP(\beta_{i}X_{in\;})}\;f(\beta /\varphi_{i})d\beta_{i}$$

where *f*(*β_i_*∣*φ_i_*) is the density function of *β_i_*, with $\varphi_{i}$ a vector of estimable parameters describing the density function (mean and variance). The simulated maximum likelihood technique is utilized to estimate the random parameters logit model using Halton draws (Train, 2009) [65]. Halton draws is more accurate than random draws [67]. The Halton numbers cover the domain of the mixing distribution more uniformly. Variation in estimated probabilities of explanatory variables over crash observations is considerably reduced using Halton draws relative to those calculated with random draws due to draws being spread more uniformly for each crash observation [68]. All models (yearly and overall model) were estimated in this study using 1000 Halton draws, which are adequate for accurate estimation of the parameters as per previous studies [52,64,69,70].

After empirically checking several statistical distributions for the density function *f*(*β_i_*∣*φ_i_*), the normal distribution was found to yield the best log-likelihood at convergence value and provided the best statistical fit to the parameter density function following previous literature [52,71]. The probabilities of significant explanatory variables leading to individual injury severity levels were calculated using marginal effects. Marginal effects indicate the effect of a unit change in the explanatory variable on the outcome of the response variable (motorcyclist injury severity). Suppose there should arise an occurrence of indicator variables, then, in that case, marginal effects represent the impact on the outcome of the response variable by switching the value of the indicator variable from 0 to 1 and vice versa.

### 3.2. Temporal Instability

Temporal instability refers to the impaired transferability of factors contributing to injury severity over the analysis period. The primary aim of this research is to statistically investigate variation in the effects of statistically significant attributes on helmet-wearing and non-helmet-wearing motorcyclists’ injury severity among different time intervals. In literature studies have observed statistically significant temporal instability in accident datasets by conducting various likelihood ratio tests [72]. The studies revealed that road user injury severity is determined by factors that change over time [59]. Significant research has revealed that temporal instability is one of the contributing factors to crash injury severity [73]. Ref. [59] discovered that the impact of highway geometric features, vehicle, and driver characteristics on resulting driver-injury severities in Chicago varied dramatically from one year to the next from 2004 to 2012. From 2010 to 2017, [74] discovered statistically significant temporal instability in injuries caused by truck crashes in Los Angeles, and [75] discovered temporal instability in Florida work zone crashes from 2012 to 2017.

A sequence of likelihood ratio tests was carried out to determine the temporal instability of the explanatory variables in the study dataset. To begin with, separate models for helmet-wearing and non-helmet-wearing motorcyclists were estimated for each year (2017, 2018, and 2019). The test statistic is as follows

*χ*^2^*_K_* = −2[*LL* (*β*_2017–2019,*K*_) − *LL* (*β*_2017,*K*_) − *LL*(*β*_2018,*K*_) − *LL*(*β*_2019,*K*_)] (5)

where *LL*(*β*_2017–2019,*K*_) is the log-probability at the combination of the rider injury-seriousness model that utilizes all information from 2017 to 2019 for rider bunch *K* (either helmet-wearing or non-helmet-wearing motorcyclists), *LL*(*β*_2017,*K*_) is the log-probability at the convergence of the model involving just 2017 information for rider group *K*, *LL*(*β*_2018, *K*_) is the log-probability at the convergence of the model involving just 2018 information for rider group *K*, and *LL*(*β*_2019, *K*_) is the log-probability at the convergence of the model involving just 2019 information for rider group *K*. For crashes including helmet-wearing motorcyclists, model evaluations gave χ^2^ value of 178.68 with 22 degrees of freedom, which were obtained by subtracting the total parameters in overall model from the sum of parameters of yearly models based on helmet-wearing motorcyclists. This χ^2^ value gives almost 99% certainty that the null hypothesis that the parameters are equivalent over these three years (2017, 2018, and 2019) can be rejected. For crashes including non-helmet-wearing motorcyclists, model evaluations gave χ^2^ of 141.06 with 26 degrees of freedom. This *χ*^2^ value gives 99% certainty that the null hypothesis that the boundaries are equivalent over these three years (2017, 2018, and 2019) can be rejected. This test suggests that significant temporal instability exists among motorcycle crashes involving motorcyclists’ compliance and defiance of helmet use as shown in Table 3. To test for temporal instability further, additional likelihood ratio tests were run [65,75]

*χ*^2^ = −2[*LL*(*β_t_*_2*t*1_) − *LL*(*β_t_*_1_)] (6)

where *LL*(*β_t_*_2*t*1_) is the log-probability at the convergence of a model containing combined parameters in light of utilizing period *t*2’s information while utilizing information from timeframe *t*1, and *LL*(*β_t_*_1_) is the log-probability at the convergence of the model utilizing timeframe *t*1’s information, with parameters as of now not confined to involving timeframe *t*2’s joined parameters, just as with the case for *LL*(*β_t_*_2*t*1_). This test was additionally turned around to such an extent that timeframe *t*1 above becomes time *t*2, and time *t*2 above becomes subset *t*1 (hence giving two test results for each model examination). The subsequent value *χ*^2^ is chi-square dispersed with degrees of freedom equal to the number of estimated parameters in the model containing converged parameters in light of utilizing timeframe *t*2’s information while utilizing information from timeframe *t*1. It tends to be utilized to decide whether the null hypothesis that the parameters are equivalent in the two periods can be rejected. The test was performed separately for yearly models of both the cases, i.e., helmet-wearing, and non-helmet-wearing motorcyclists. Involving the combined parameters of the 2018 model for helmet-wearing motorcyclists (Table 4) as beginning values and applying them to the 2017 information gave *χ*^2^ = 1583.938 (from Equation (6)). Twelve degrees of freedom gave a *χ*^2^ certainty level of close to 100% that the null hypothesis that the two periods are the equivalent can be rejected. Involving the converged parameters of the 2019 model for helmet-wearing motorcyclists (Table 4) as beginning values and applying them to the 2017 information gave *χ*^2^ = 728.024. With 14 degrees of freedom, this likewise gave a *χ*^2^ certainty level of close to 100% that the null hypothesis that the two periods are the equivalent can be dismissed. Additionally, different times of interest were tried for temporal stability for both cases (Table 4 and Table 5). All tests showed that the null hypothesis that the parameters were equivalent among the years could be rejected, shown in Table 3. 

## 4. Results and Discussion

### 4.1. Helmet Users

Table 6, Table 7 and Table 8 show the model estimation results for helmet-wearing motorcyclists for 2017, 2018, and 2019. The models have a reasonably good overall statistical fit, with ρ2 values of 0.576, 0.567, and 0.528 for 2017, 2018, and 2019, respectively. All the models and marginal effects were estimated using 1000 Halton draws in NLOGIT software. As demonstrated in Table 3, Table 4, Table 5, Table 6, Table 7 and Table 8, several parameters in each of the six estimated models were random and normally distributed. In the 2017 model (Table 6), the “major arterial road indicator” was a statistically significant random parameter with statistically significant heterogeneity in mean and variance. The mean of this variable varied with a large truck indicator. This indicates that the probability of fatal injuries to helmet-wearing motorcyclists on major arterial roads increased if a large truck was involved in a crash. The variance of this variable is a function of the riders aged between 20 to 30 years. In the 2018 model (Table 7), the weekday indicator was a statistically significant random parameter with heterogeneity in mean and variance. The mean of this variable decreased due to the riders aged above 50 years indicator. Hence, there is less likelihood of severe injuries to above 50 years old helmet-wearing motorcyclists on a weekday. The variance of the random parameter was a function of the passenger car indicator, with crashes involving a collision with a passenger car likely to have a higher variance. In the 2019 model (Table 8), the below 20-year-old indicator produced a statistically significant random parameter with statistically significant heterogeneity in mean and variance. It was found that the mean of the random parameter increased with crashes that occurred due to over-speeding. Hence, there is an improved probability of severe injuries in crashes involving over-speeding helmet-wearing motorcyclists aged below 20 years. The variance was a component of the distracted driving indicator, with crashes involving distracted crashes likely to have a lower variance. Possible causes of distraction could be cell phone use while bike riding, looking at billboards, or talking with a pillion rider.

### 4.2. Non-Helmet Users

Table 9, Table 10 and Table 11 show the model estimation results for non-helmet-wearing motorcyclists in 2017, 2018, and 2019 respectively. The models had substantially lower goodness of fit than their helmet-wearing partners, with ρ^2^ values of 0.504, 0.458, and 0.405 for 2017, 2018, and 2019. In the 2017 model (Table 9), the “peak hour” indicator produced a random parameter with heterogeneity in mean and variance. The random variable’s mean increased if the motorcyclists’ age was in the range from 20 to 30 years. Hence, there is an improved probability of severe injuries for non-helmet-wearing motorcyclists aged in the range from 20 to 30 years riding a bike during peak traffic hours (morning peak = 7 a.m. to 10 a.m., afternoon peak = 1 p.m. to 3 p.m., evening peak = 6 p.m. to 9 p.m.). The variance was a component of the over-speeding indicator, with crashes due to over-speeding likely to have a lower variance. In the 2018 model (Table 10), the over-speeding indicator was a statistically significant random parameter with heterogeneity in mean and variance. The random variable’s mean decreased if the motorcyclists’ age was below 20 years. Hence, there is less likelihood of severe injuries to over-speeding non-helmet-wearing motorcyclists below 20 years. The large truck indicator was found to increase the variance of random parameters. In the 2019 model (Table 11), the weekday indicator was viewed as a significant random parameter with heterogeneity in mean and variance. It was found that the mean of the random variable increased with the over-speeding indicator. This indicates an improved probability of severe injuries to non-helmet-wearing motorcyclists in the case of over-speeding on weekdays. The variance of the random parameter was found to be a function of the U-turn indicator, with crashes that occurred due to a U-turn likely to be of higher variance.

### 4.3. Effect of Explanatory Variables

All six models in Table 6, Table 7, Table 8, Table 9, Table 10 and Table 11 include a wide scope of roadway, crash, rider, vehicle, traffic, and temporal attributes affecting the motorcyclists’ injury severities. Table 12 displays the marginal effects of all genuinely critical factors for helmet-wearing and non-helmet-wearing motorcyclists’ crashes by year and injury level to compare every one of the six models.

#### 4.3.1. Roadway Attributes

In the 2017 model, the lane 2 indicator was significantly associated with a minor injury, meaning that motorcycle crashes happening on two-lane side roads are more likely to cause minor injuries to helmet-wearing motorcyclists. This result was not statistically significant for the 2018 and 2019 models for helmet-wearing motorcyclists, and for non-helmet-wearing motorcyclists, the lane 2 indicator was significantly associated with a minor injury, meaning that motorcycle crashes happening on two-lane side roads are more likely to cause severe injuries. The lane 3 indicator was only significant in severe injuries in helmet-wearing motorcyclists. Motorcycle crashes occurring on collector roads were found to have a lower likelihood of severe injuries and a higher probability of minor injuries to helmet-wearing motorcyclists for the 2017 model only, while they had a higher probability of severe injuries on the 2019 non-helmet-wearing model.

#### 4.3.2. Rider Attributes

The motorcyclists’ ages were classified into different age groups under the rider attributes category. The below 20 years old indicator was significant in the severe injury category in the 2017 and 2018 models for helmet-wearing motorcyclists, and the 2018 and 2019 models were significant in terms of severe injuries and fatalities for non-helmet-wearing motorcyclists. Motorcyclists aged between 20 and 30 years were found to be highly likely to be caused minor injuries in 2017 helmet and non-helmet-wearing models, while the probability of fatal injury was higher for non-helmet-wearing motorcyclists aged between 40 and 50 years in the 2017 model only. Helmet-wearing motorcyclists above 50 years were highly likely to be caused fatal injuries in the 2018 and 2019 models. This is consistent with the past findings. Riders aged 26–39 are more prone to suffer severe and fatal injuries [76]. The results are similar to the study [62], in which the author argued that the old and middle-aged motorcyclists indicators produced temporal instability across the 2005–2015 crash data. A study discovered that riders aged 19–55 years had more severe crash injuries than younger drivers [77]. Higher injury severity for riders aged 25–50 years may be attributed to greater risk exposure during the morning and evening rush hours since they are primarily people who work. This conclusion is consistent with previous studies [78,79] since older motorcyclists take longer to respond to hazardous situations and their physiological state deteriorates. For non-helmet-wearing motorcyclists, this variable was significant for fatal injuries in the 2018 and 2019 models. Females as pillion rider indicators show that female pillion riders were likely to suffer from severe injuries in helmet-wearing motorcyclists’ crashes in 2018 and 2019. This finding is similar to an earlier study conducted in Pakistan that revealed that female pillion passengers’ risk of fatality or severe injuries increased by 3% if their clothes were trapped in the wheels [80]. The increased likelihood of this kind of crash is due to the fact that females in Pakistan typically wear loose garments such as long, flowing overcoats (abayas/burqas) or long shirts (kameez) over trousers (shalwar). Additionally, they sit on the motorcycle in a side-ways manner. Therefore, their clothes can easily become trapped in the chain or rear wheel of the motorcycle, making the rider and passenger more susceptible to suffering fatal injuries [81,82]. Being a male rider was significant for minor injuries in the 2017 helmet-wearing model and significant for severe injuries in the 2017 non-helmet-wearing model only.

#### 4.3.3. Crash Characteristics

For variables related to crash or vehicle characteristics, the passenger car indicator shows that motorcycle collisions with passenger cars had a higher likelihood of minor injuries to helmet-wearing motorcyclists for the 2018 and 2019 models and a higher probability of fatalities for the 2017 model. For non-helmet-wearing motorcyclists, collisions with passenger cars were more likely to cause severe injuries in 2018 and minor injuries in 2019. The results are consistent with the previous findings of motorcycle crashes in the same region of Pakistan. This finding might be due to the high speeds of cars leading to more severe injuries [64]. The motorcycle indicator shows that a collision of two motorcycles reduces the probability of fatal injuries for helmet-wearing motorcyclists for 2018 and is more likely to cause fatal injuries to the motorcyclists in the 2019 model. In contrast, for non-helmet-wearing motorcyclists, a collision of two motorcycles decreases the probability of minor injury and increases the chances of severe injuries for the 2019 model. The large truck indicator shows that motorcycle collisions with large trucks would bring about minor injuries and severe injuries to helmet-wearing motorcyclists for the 2017 and 2019 models, respectively. The same indicator is significant in severe injuries to non-helmet-wearing motorcyclists in the 2017 and 2018 models. The results are in agreement with the finding from a previous study [79], where the author stated that the collision of a motorcycle with a truck might lead to a higher probability of severe injuries. In Pakistan, the auto-rickshaw indicator, known as “qingqi”, was significant only in the 2017 model for non-helmet-wearing motorcyclists. Collisions with qingqi were found to be more likely to result in no injury to the motorcyclists than severe and fatal injuries. This finding is intuitive due to the relatively lesser speed and weight characteristics of the rickshaw. According to earlier research, the height, weight, and size of the vehicles involved can have a substantial impact on the severity of the injuries resulting from a crash [83,84]. An earlier study conducted in Pakistan showed a similar result.

#### 4.3.4. Violation Attributes

The wrong-way indicator was found to be significant in the no injury category in the 2017 model for the non-helmet-wearing model. Distraction was a significant indicator in all six models for crashes involving helmet-wearing and non-helmet-wearing motorcyclists. This indicator shows that fatal injury is high for both helmet-wearing and non-helmet-wearing motorcyclists in crashes caused by distraction in all models. Distracted driving is dangerous, claiming 3142 lives in 2019 [85]. Distractions may include using a cell phone while bike riding, looking at advertising boards, or talking with pillion riders. The U-turn indicator was the only significant indicator across all years for non-helmet-wearing motorcyclists and was found to be significant in 2017 and 2019 for helmet-wearing motorcyclists. All models for non-helmet-wearing motorcyclists warranted a high probability of no injury, severe injury, and fatalities in 2017, 2018, and 2019, respectively. For helmet-wearing motorcyclists, the likelihood of minor injury and severe injury was higher due to U-turns compared to fatal injuries. A previous study conducted in the context of Pakistan found that the U-turn indicator also increases the risk of fatal injuries by 3% in single vehicle motor crashes [80]. This could be explained by the fact that motorcyclists in Pakistan frequently turn around in unusual places. As a result, they are more likely to be exposed to numerous hazardous situations and be involved in side-impact collisions with oncoming cars, which can result in fatal motorcycle crashes [86]. In contrast, the over-speeding indicator was the significant indicator across all analysis years for helmet-wearing motorcyclists, while it was found to be significant in the 2017 and 2019 models for non-helmet-wearing motorcyclists. In all cases where over-speeding was significant, over-speeding had a higher likelihood of minor injuries as compared to all other injury categories.

#### 4.3.5. Temporal Attributes

The off-peak hour indicator under the temporal variables category was only a significant variable in the 2017 model for non-helmet-wearing motorcyclists. This shows that crashes occurring during off-peak hours were less likely to result in fatal injuries than other injury categories, as shown in Table 8. The weekday indicator was found to be significant in the 2017 and 2018 models for non-helmet-wearing motorcyclists and was not found to be significant in any models for helmet-wearing motorcyclists. The weekday indicator shows a high likelihood of fatal injuries and minor injuries to motorcyclists for both models. The findings are not in accord with the previous finding, which interpreted that the weekday indicator was found to be less likely to cause fatal injuries [80]. The spring indicator was only found to be significant in the 2019 model for non-helmet-wearing motorcyclists and was not found to be significant in any of the models for helmet-wearing motorcyclists. The spring indicator was found to be highly likely to cause no injury to the motorcyclists owing to favorable weather and bike-riding conditions. Results of temporal instability in models for helmet-wearing and non-helmet-wearing motorcyclists are presented in Table 3. 

**Table 12:** Comparison of marginal effects in models for helmet-wearing and non-helmet-wearing motorcyclists throughout the long term (minimal impacts for non-helmet-wearing motorcyclists in parenthesis).

Variables	No Injury			Minor Injury			Severe Injury			Fatal Injury		
	2017	2018	2019	2017	2018	2019	2017	2018	2019	2017	2018	2019
**Roadway attributes**												
Lane 2 indicator	−0.0017 --	-- (−0.0010)	-- --	0.0009 --	-- (0.0003)	-- --	0.0005 --	-- (0.0005)	-- --	0.0003 --	-- (0.0002)	-- --
Lane 3 indicator	−0.0007 --	-- --	-- --	−0.0123 --	-- --	-- --	0.0149 --	-- --	-- --	−0.0019 --	-- --	-- --
Collector road indicator	0.0007 --	-- --	-- (0.0027)	0.0041 --	-- --	-- (−0.0082)	−0.0065 --	-- --	-- (0.0029)	0.0017 --	-- --	-- (0.0026)
**Rider Attributes**												
Below 20 years indicator	−0.0006 --	−0.0004 (−0.0010)	(−0.0020)	−0.0159 --	−0.0078 (−0.0102)	(-0.0131)	0.0208 --	0.0116 (−0.0085)	(0.0177)	−0.0043 --	−0.0033 (0.0193)	(-0.0026)
Between 20–30 indicator	0.0016 (−0.0030)	--	--	0.0118 (0.0328)	--	--	0.0080 (−0.0190)	--	--	−0.0214 (−0.0108)	--	--
Between 40–50 indicator	-- (−0.0002)	-- --	-- --	-- (−0.0029)	-- --	-- --	-- (−0.0011)	-- --	-- --	-- (0.0042)	-- --	-- --
Above 50 Years indicator	-- --	−0.0006 (−0.0006)	−0.0005 (−0.0010)	-- --	−0.0131 (−0.0102)	−0.0114 (−0.0124)	-- --	−0.0038 (−0.0085)	−0.0050 (−0.0049)	-- --	0.0175 (0.0193)	0.0169 (0.0184)
Female Indicator	-- --	−0.0008 --	−0.0020 --	-- --	−0.0342 --	−0.0586 --	-- --	0.0520 --	0.0858 --	-- --	−0.0170 --	−0.0252 --
Male indicator	−0.0529 (−0.0175)	-- --	-- --	0.5925 (−0.3261)	-- --	-- --	−0.3533 (0.3686)	-- --	-- --	−0.1863 (−0.0250)	-- --	-- --
**Crash attributes**												
Passenger car indicator	−0.0001	0.0006 (0.0002)	0.0006 (0.0003)	−0.0014	0.0028 (0.0007)	0.0048 (0.0016)	−0.0008	0.0004 (0.0011)	0.0008 (0.0003)	0.0024	−0.0038 (−0.0019)	−0.0062 (−0.0022)
Motorcycle indicator		−0.0012 --	0.0092 (0.0270)	--	0.0080 --	−0.0722 (−0.0870)	--	−0.0030 --	0.0267 (0.0308)	--	−0.0038 --	0.0363 (0.0292)
Large truck	−0.0003 (−0.0001)	-- (0.0001)	0.0001 --	−0.0040 (−0.0016)	-- (0.0001)	0.0011 --	0.0047 (0.0017)	-- (0.0004)	0.0002 --	−0.0003 (−0.0001)	-- (−0.0006)	−0.0014 --
Auto-rickshaw indicator	-- (0.0021)	-- --	-- --	-- (−0.0016)	-- --	-- --	-- (−0.0004)	-- --	-- --	-- (−0.0001)	-- --	-- --
**Violation Attributes**												
Wrong-way indicator	-- (0.0018)	-- --	-- --	-- (−0.0052)	-- --	-- --	-- --	-- (0.0025)	-- --	-- (0.0009)	-- --	-- --
Distraction indicator	0.0025 (−0.0061)	−0.0055 (−0.0038)	−0.0055 (−0.0105)	−0.0555 (−0.2093)	0.0015 (0.0012)	0.0016 (0.0039)	0.0147 (−0.0507)	0.0006 (0.0014)	0.0010 (0.0017)	0.0382 (0.2661)	0.0034 (0.0012)	0.0029 (0.0049)
U-turn indicator	0.0002 (.0054)	-- (0.0001)	−0.0016 (0.0003)	0.0010 (−0.0173)	-- (0.0003)	0.0056 (0.0002)	0.0007 (0.0088)	-- (0.0004)	−0.0014 (0.0001)	−0.0019 (0.0031)	-- (−0.0007)	−0.0026 (−0.0006)
Over-speeding indicator	0.0027 (−0.0029)	−0.0402 --	−0.0384 (−0.1287)	0.0274 (−0.0910)	0.2110 --	0.2490 (0.3260)	0.0144 (0.0953)	−0.0815 --	−0.1077 (−0.1361)	−0.0445 (−0.0015)	−0.0894 --	−0.1029 (−0.0611)
**Temporal attributes**												
Off-peak hour indicator	-- (0.0003)	-- --	-- --	-- (0.0075)	-- --	-- --	-- (0.0020)	-- --	-- --	-- (−0.0098)	-- --	-- --
Weekday indicator	-- (−0.0007)	-- (0.0020)	-- --	-- (−0.0167)	-- (0.0160)	-- --	-- (−0.0043)	-- (−0.0293)	-- --	-- (0.0218)	-- (0.0112)	-- --
Spring indicator	-- --	-- --	-- (0.0101)	-- --	-- --	-- (−0.0081)	-- --	-- --	-- (−0.0014)	-- --	-- --	-- (−0.0006)

## 5. Conclusions

Motorcycle safety is affected by an unwillingness to wear a helmet, non-standard helmets, the desire to speed, running a red light, underage riding, and an absence of a requirement of road safety regulations by the concerned agencies. This study attempted to find various key risk factors influencing the injuries sustained by helmet-wearing and non-helmet-wearing motorcyclists in motorcycle crashes in a developing country, Pakistan. Using motorcycle crash data from 2017 to 2019 in Rawalpindi city, this study employed a random parameters logit model with heterogeneity in means and variances. Four injury levels were considered: no injury, minor injury, severe injury, and fatal injury. Critical variables included in the study, consisting of rider characteristics, roadway attributes, violations, and temporal characteristics, had a considerable impact on motorcyclist injury severity outcomes. There were notable discrepancies between crashes involving helmet-wearing and non-helmet-wearing motorcyclists. Both helmet-wearing and non-helmet-wearing motorcyclists’ crashes exhibited temporal instability over the three-year analysis period considered in this study. This is a significant finding. The model assessment result for helmet-wearing and non-helmet-wearing motorcyclists, accounting for temporal instability, can potentially help guide injury severity mitigation policies.

The models for helmet-wearing motorcyclists had noticeably superior goodness of fit than non-helmet-wearing motorcyclists for all three years. All six models showed significant heterogeneity in the random parameters’ mean and variance for helmet and non-helmet motorcyclist crashes. Model estimates suggest significant variations in the impact of explanatory variables on motorcyclists’ injury severity in the case of compliance with and defiance of helmet use. For helmet-wearing motorcyclists, the key factors young riders (below 20 years of age), female pillion riders, collisions with another motorcycle, crashes due to over-speeding, passenger car indicators, 50 years and above drivers, and distracted driving were significantly associated with increasingly severe injuries and fatal injuries. In contrast, for non-helmet-wearing motorcyclists, the significant factors responsible for severe injuries and fatalities were distracted driving, collision of two motorcycles, U-turns crashes, weekday crashes, drivers above 50 years, and over-speeding. Future research that can identify temporally stable and unstable subsets of the crash population could be very useful in influencing safety policy.

In light of the existing findings, some immediate motorcycle safety countermeasures are hereby suggested to improve the safety of helmet-wearing and non-helmet-wearing motorcyclists. Firstly, for helmet-wearing motorcyclists, considering the high possibility of undergoing severe injuries by riders below 20 years of age, it is necessary to design and conduct road safety awareness campaigns to raise awareness about the importance of cautious driving. Moreover, female pillion riders need to be educated not to wear loose clothes with trailing ends while sitting on a bike as a pillion rider. It is recommended to build adequate covers for the motorbike’s back wheel and chains to avoid female clothing from being trapped in the motorcycle. In Pakistan, it is usually quite normal during congested traffic for a motorbike find their way in between the traffic stream or to use a pedestrian walk to cross the blocked area. To counter this issue, separate lanes should be added/designed for motorcyclists so they can ride smoothly and safely. Violating laws such as making U-turns at unspecified locations, which can result in severe injuries, should be strictly fined. As far as the non-helmet-wearing motorcyclists are concerned, the findings that crashes caused by distraction are more vulnerable to severe and fatal injuries warrant additional consideration to policymakers and city traffic police. Such crashes may be caused by cell phone distraction. Heavy fines should be imposed on the use of cell phones while bike riding. High-visibility enforcement (HVE) measures should be adopted for distracted driving to reduce cell phone use while driving. Educating riders and making them aware of the factors found to increase the severity of injuries, as well as the appropriate enforcement and regulation of traffic laws, particularly those pertaining to motorcycle registration and driving licensing, may be useful in minimizing risks and reducing the number of road crash injuries and fatalities involving motorcycles.

This is the first study of its kind in Pakistan aiming to assess the risk factors affecting the helmet-wearing and non-helmet-wearing motorcyclists’ injury severities through statistical model estimation. It should be noted that this paper has several limitations. Only temporal instability was considered in this study; however, spatial instability could be a practical problem that should be explored more in the future. As well as, how the risk factors associated with motorcyclists interact with other contributing factors such as riders’ mental and emotional well-being, route choice, adverse roadway geometry, and even some social attributes such as the level of bike ownership, monthly income of the bike rider, etc., should be considered. The study results are expected to motivate all relevant stakeholders to improve overall traffic safety in general and motorcycle safety in particular in the country.

## Figures and Tables

**Table 1:** Summary of previous methodological approaches used for helmet-wearing and non-helmet-wearing motorcyclists’ injury severities.

Methodology Adopted	Previous Research
Logistic regression	[41,42,43]
Finite element head model	[44]
Univariate analysis and multiple logistic regression	[45]
Latent class cluster and random parameters logit model	[46]
Student’s *t*-test and Pearson’s χ^2^ *p*-value	[25]
Partial proportional odds model	[47]
Cross-sectional observational study	[48,49]
Retrospective study	[25,50]
Fixed effects regression models	[51]

**Table 2:** Descriptive statistics of significant variables in the helmet-wearing and non-helmet-wearing motorcyclists’ injury severity models.

Variable	2017				2018				2019			
	Helmet		No Helmet		Helmet		No Helmet		Helmet		No Helmet	
	Mean	S.D.	Mean	S.D.	Mean	S.D.	Mean	S.D.	Mean	S.D.	Mean	S.D.
**Roadway Attributes**												
Major arterial indicator (1 if crash occurred on major arterial, 0 otherwise)	0.597	0.490	0.640	0.479	0.590	0.496	0.607	0.488	0.209	0.497	0.523	0.499
Lane 2 indicator (1 if crash occurred on two lanes each side road, 0 otherwise)	0.212	0.409	0.166	0.372	0.166	0.372	0.158	0.365	0.152	0.359	0.143	0.350
Local road indicator (1 if crash occurred on local road, 0 otherwise)	0.054	0.226	0.059	0.236	0.044	0.205	0.046	0.249	0.046	0.241	0.046	0.210
Collector road indicator (1 if crash occurred on collector road, 0 otherwise)	0.142	0.349	0.119	0.324	0.154	0.361	0.144	0.353	0.195	0.396	0.219	0.414
Speed: 60 kmph indicator (1 if crash occurred at a road with speed limit 60 kmph, 0 otherwise)	0.645	0.478	0.674	0.468	0.697	0.459	0.690	0.462	0.747	0.434	0.722	0.447
**Violation Attributes**												
Wrong-way indicator (1 if crash occurred while riding the wrong way, 0 otherwise)	0.005	0.076	0.009	0.095	0.010	0.101	0.012	0.109	0.019	0.139	0.010	0.100
Distraction indicator (1 if crash occurred due to distraction, 0 otherwise)	0.272	0.445	0.269	0.448	0.245	0.430	0.231	0.421	0.236	0.425	0.215	0.410
U-turn indicator (1 if crash occurred on U-turn, 0 otherwise)	0.030	0.172	0.032	0.176	0.027	0.162	0.032	0.176	0.033	0.179	0.046	0.211
Over-speeding indicator (1 if crash occurred due to over-speeding, 0 otherwise)	0.690	0.462	0.688	0.430	0.716	0.450	0.724	0.446	0.709	0.453	0.727	0.445
**Rider attributes**												
Between 20–30 years indicator (1 if motorcyclist’s age lies between 20 and 30 years, 0 otherwise)	0.354	0.478	0.397	0.489	0.377	0.484	0.395	0.485	0.361	0.480	0.350	0.477
Male indicator (1 if motorcyclist was male, 0 otherwise)	0.877	0.328	0.865	0.341	0.822	0.322	0.832	0.343	0.867	0.331	0.878	0.324
Below 20 years indicator (1 if motorcyclist’s age is below 20 years, 0 otherwise)	0.208	0.411	-	-	0.185	0.388	0.210	0.407	0.203	0.402	0.263	0.440
Between 40–50 indicator (1 if motorcyclist’s age lies between 40–50 years, 0 otherwise)	0.226	0.418	0.103	0.304	0.118	0.323	0.107	0.310	0.118	0.322	0.101	0.302
**Crash Attributes**												
Large truck indicator (1 if crash occurred with truck, 0 otherwise)	0.059	0.236	0.019	0.143	0.060	0.238	0.022	0.147	0.815	0.387	0.013	0.118
Motorcycle indicator (1 if two motorcycles collided with each other, 0 otherwise)	0.132	0.339	0.142	0.349	0.141	0.348	0.157	0.364	0.056	0.230	0.832	0.378
Passenger car indicator (1 if crash occurred with passenger car, 0 otherwise)	0.165	0.371	0.169	0.375	0.162	0.368	0.166	0.372	0.164	0.370	0.177	0.382
Auto-rickshaw indicator (1 if crash occurred with auto-rickshaw, 0 otherwise)	0.724	0.236	0.041	0.198	0.040	0.197	0.049	0.217	0.041	0.199	0.052	0.223
**Temporal Attributes**												
Weekday indicator (1 if crash occurred on weekday, 0 otherwise)	0.711	0.452	0.718	0.449	0.708	0.454	0.709	0.451	0.717	0.450	0.720	0.448
Weekend indicator (1 if crash occurred on weekend, 0 otherwise)	0.288	0.452	0.281	0.449	0.291	0.454	0.209	0.454	0.282	0.450	0.279	0.448
Off-peak hour indicator (1 if crash occurred in off-peak hours, 0 otherwise)	0.494	0.499	0.500	0.500	0.501	0.500	0.512	0.499	0.508	0.499	0.504	0.499
Spring indicator (1 if crash occurred in spring season, 0 otherwise)	0.158	0.365	0.171	0.376	0.170	0.376	0.157	0.363	0.152	0.359	0.164	0.371

**Table 3:** Results of temporal instability for helmet-wearing and non-helmet-wearing motorcyclists.

Model Goodness of Fit Values	Model for Motorcyclists Wearing Helmet	Model for Motorcyclists Not Wearing Helmet
Log-likelihood at convergence of overall model *LL*(*β*_2017–2019_)	−4025.185	−13,641.844
Log-likelihood at convergence of 2017 model *LL*(*β*_2017_)	−1199.931	−3477.439
Log-likelihood at convergence of 2018 model *LL*(*β*_2018_)	−1258.448	−4151.247
Log-likelihood at convergence of 2019 model *LL*(*β*_2019_)	−1477.467	−5942.630
*χ*^2^ Value	178.68	141.06
Degrees of Freedom	22	26
Level of Confidence	99%	99%
Critical χ^2^ value	40.29	45.64
Conclusion	**Temporally unstable**	**Temporally unstable**

**Table 4:** Likelihood ratio test results between different periods based on a random parameter approach with heterogeneity in means and variances in helmet-wearing motorcyclists’ crash data (χ^2^ values with degrees of freedom in parenthesis and confidence level in brackets).

*t*_1_	*t*_2_		
	2017	2018	2019
2017	-	1583.938 (12) [>99%]	728.024 (14) [>99%]
2018	198.152 (17) [>99%]	-	83.33 (14) [>99%]
2019	305.584 (17) [>99%]	25.402 (12) [>99%]	-

**Table 5:** Likelihood ratio test results between different periods based on a random parameter approach with heterogeneity in means and variances in non-helmet-wearing motorcyclists’ crash data (χ^2^ values with degrees of freedom in parenthesis and confidence level in brackets).

*t*_1_	*t*_2_		
	2017	2018	2019
2017	-	1738.202 (15) [>99%]	1446.054 (14) [>99%]
2018	1859.062 (19) [>99%]	-	121.158 (14) [>99%]
2019	9360.034 (19) [>99%]	4870.566 (15) [>99%]	-

**Table 6:** Model estimation results for helmet-wearing motorcycle crashes 2017.

Variables	Parameter Estimates	*t*-Stats	Marginal Effects			
			No Injury	Minor Injury	Severe Injury	Fatal Injury
Constant [MI]	−1.536	−2.84				
Constant [SI]	1.526	8.81				
Constant [FI]	3.267	14.71				
Random parameter (normally distributed)						
Major arterial [FI]	0.367	1.87	−0.0009	−0.0167	−0.0046	0.0222
Heterogeneity in the mean of the random parameter						
Large truck (1 if a crash occurred with a truck, 0 otherwise) [SI]	−1.783	−2.37				
Heterogeneity in variance of the random parameter						
Between 20–30 years (1 if motorcyclist’s age lies between 20 and 30 years, 0 otherwise) [SI]	0.581	1.91				
Roadway Attributes						
Lane 2 indicator (1 if a crash occurred on two-lane each side road, 0 otherwise) [NI]	−1.736	−2.38	−0.0017	0.0009	0.0005	0.0003
Collector road (1 if an accident happened on a collector road, 0 in any case) [SI]	−0.672	−2.47	0.0007	0.0041	−0.0065	0.0017
Rider Attributes						
Between 20–30 years indicator ( 1 if motorcyclists age is between 20 and 30, 0 otherwise) [FI]	−1.292	−5.62	0.0016	0.0118	0.0008	−0.0214
Below 20 years indicator (1 if that motorcyclist’s age is under 20 years, 0 in any case) [SI]	0.75	4.38	−0.0006	−0.0159	0.0208	−0.0043
Male rider (1 if a male rider was involved in crash only, 0 otherwise) [MI]	5.487	10.44	−0.0529	0.5925	−0.3533	−0.1863
Crash Attributes						
Passenger car indicator (1 if an accident happened with a Passenger car, 0 otherwise) [FI]	−1.851	−2.96	−0.0001	−0.0014	−0.0008	0.0024
Large Truck indicator (1 if an accident happened with a truck, 0 in any case) [SI]	0.584	2.09	−0.0003	−0.0004	0.0047	−0.0003
Violation Attributes						
Distraction indicator (1 if an accident happened due to distraction, 0 otherwise) [MI]	−1.481	−8.03	0.0025	−0.0555	0.0147	0.0382
U-Turn indicator (1 if an accident happened at U-turn, 0 otherwise) [FI]	−4.147	−4.01	0.0002	0.0001	0.0007	−0.0019
Over-speeding indicator (1 if an accident happened due to over-speeding, 0 otherwise) [FI]	−3.685	−9.67	0.0027	0.0274	0.0144	−0.0445
Number of observations	2045					
Number of parameters	17					
Log-likelihood at zero	−2834.971					
Log-likelihood at convergence	−1199.931					
ρ^2^ = 1 − *LL*(*β*)/*LL*(0)	0.576					

**Table 7:** Model estimation results for helmet-wearing motorcycle crashes 2018.

Variables	Parameter Estimates	*t*-Stats	Marginal Effects			
			No Injury	Minor Injury	Severe Injury	Fatal Injury
Constant [FI]	0.886	8.41				
Random parameter (normally distributed)						
Weekday indicator [SI]	−3.72	−6.94	−0.0005	−0.0263	0.0204	0.0064
Heterogeneity in the mean of the random parameter						
Above 50 years indicator (1 if motorcyclist’s age is above 50 years, 0 otherwise) [FI]	−1.353	−1.71				
Heterogeneity in the variance of random parameter						
Passenger car indicator (1 if an accident happened with a passenger car, 0 otherwise) [FI]	0.201	1.78				
Rider Attributes						
Female indicator (1 if pillion rider involved was female, 0 otherwise) [SI]	5.539	22.37	−0.0008	−0.0342	0.0052	−0.017
Below 20 years indicator (1 if motorcyclist’s age is under 20 years, 0 otherwise) [SI]	1.13	4.71	−0.0004	−0.0078	0.0116	−0.0033
Above 50 years indicator (1 if motorcyclist’s age is above 50 years, 0 otherwise) [FI]	1.505	5.33	−0.0006	−0.0131	−0.0038	0.0175
Crash Attributes						
Motorcycle indicator (1 if two motorcyclists collide with each other, 0 otherwise) [MI]	0.952	2.99	−0.0012	0.0008	−0.0003	−0.0038
Passenger car indicator (1 if an accident happened with a passenger car, 0 otherwise) [FI]	−3.057	−5.33	0.0006	0.0028	0.0004	−0.0038
Violation Attributes						
Distraction indicator (1 if an accident happened due to distraction, 0 otherwise) [NI]	−3.198	−6.3	−0.0055	0.0015	0.0006	0.0034
Over-speeding indicator (1 if an accident happened due to over-speeding, 0 otherwise) [MI]	3.825	25.48	−0.0402	0.211	−0.0815	−0.0894
Number of observations	2095					
Number of parameters	12					
Log-probability at zero	−2904.287					
Log-probability at union	−1258.448					
ρ^2^ = 1 − *LL*(β)/*LL*(0)	0.567					

**Table 8:** Model estimation results for helmet-wearing motorcycle crashes 2019.

Variables	Parameter Estimates	*t*-Stats	Marginal Effects			
			No Injury	Minor Injury	Severe Injury	Fatal Injury
Constant [MI]	1.262	5.71				
Constant [FI]	1.248	9.29				
Random parameter (normally distributed)						
Below 20-year indicator [SI]	−1.861	−3.17	−0.0009	−0.0326	0.0345	−0.0011
Heterogeneity in the mean of the random parameter						
Over-speeding indicator (1 if a crash occurred due to over-speeding, 0 otherwise) [MI]	3.439	6.45				
Heterogeneity in the variance of the random parameter						
Distraction indicator (1 if a crash occurred due to distraction, 0 otherwise) [NI]	0.883	1.78				
Rider Attributes						
Female (1 on the off chance that pillion rider included was female, 0 otherwise) [SI]	3.63	23.94	−0.002	−0.0586	0.0858	−0.0252
Above 50 years indicator (1 if motorcyclist’s age is above 50 years, 0 otherwise) [FI]	0.929	5.14	−0.0005	−0.0114	−0.005	0.0169
Crash Attributes						
Motorcycle indicator (1 if two motorcycles collide with each other, 0 otherwise) [MI]	−0.617	−3.49	0.0092	−0.0722	0.0267	0.0363
Passenger car (1 if a crash occurred with a passenger car, 0 otherwise) [FI]	−2.44	−5.82	0.0006	0.0048	0.0008	−0.0062
Large truck (1 if a crash occurred with a truck, 0 otherwise) [MI]	−3.259	−3.28	0.0001	0.0011	0.0002	−0.0014
Violation Attributes						
U-turn indicator (1 if crash occurred at U-turn, 0 otherwise) [MI]	0.738	2.61	−0.0016	0.0056	−0.0014	−0.0026
Over-speeding indicator (1 if a crash occurred due to over-speeding, 0 otherwise) [MI]	2.801	17.89	−0.0384	0.0249	−0.1077	−0.1029
Distraction indicator (1 if a crash occurred due to distraction, 0 otherwise) [NI]	−2.516	−5.4	−0.0055	0.0016	0.0001	0.0029
Number of perceptions	2262					
Number of parameters	14					
Log-likelihood at zero	−3135.797					
Log-likelihood at convergence	−1477.467					
ρ^2^ = 1 − *LL*(*β*)/*LL*(0)	0.528					

**Table 9:** Model estimation results for non-helmet-wearing motorcycle crashes 2017.

Variables	Parameter Estimates	*t*-Stats	Marginal Effects			
			No Injury	Minor Injury	Severe Injury	Fatal Injury
Constant [MI]	3.369	24.73				
Constant [SI]	4.769	23.9				
Constant [FI]	−0.831	−3.9				
Random parameter (normally distributed)						
Peak hour indicator [SI]	−0.659	−3.92	0.0000	−0.0016	0.0045	−0.003
Heterogeneity in the mean of the random parameter						
Between 20–30 years indicator (1 if motorcyclist’s age is between 20 and 30 years, 0 otherwise) [MI]	0.533	3.33				
Heterogeneity in the variance of the random parameter						
Over-speeding indicator (1 if a crash occurred due to over-speeding, 0 otherwise] [SI]	−0.726	−2.59				
Rider Attributes						
Between 20–30 years indicator (1 if motorcyclist’s age lies b/w 20 and 30 years, 0 otherwise) [MI]	0.534	6.14	−0.0003	0.0328	−0.019	−0.0108
Between 40–50 years indicator (1 if motorcyclist’s age lies b/w 40 and 50 years, 0 in any case) [FI]	0.518	3.08	−0.0002	−0.0029	−0.0011	0.0042
Male indicator (1 if a male rider was involved in crash, 0 otherwise)	−4.097	−24.31	0.0175	0.3261	0.3686	0.025
Roadway attributes						
Lane 3 indicator (1 if a crash happened on 3 lane road, 0 otherwise) [SI]	0.196	2.04	−0.0007	−0.0123	0.0149	−0.0019
Crash Attributes						
Auto-rickshaw indicator (1 if a crash happened with auto-cart, 0 otherwise) [NI]	0.936	2.79	0.0021	−0.0016	−0.0004	−0.0001
Large truck indicator (1 if an accident happened with a truck, 0 otherwise) [SI]	0.571	2.22	−0.0001	−0.0016	0.0017	−0.0001
Violation Attributes						
Wrong-way indicator (1 if an accident happened the wrong way, 0 otherwise) [MI]	−3.346	−9.32	0.0018	−0.0052	0.0025	0.0009
Distraction indicator (1 if an accident happened due to distraction, 0 otherwise) [FI]	4.48	30.68	−0.0061	−0.2093	−0.0507	0.2661
U-turn indicator (1 if an accident happened at U-turn, 0 otherwise) [MI]	−2.596	−12.88	0.0054	−0.0173	0.0088	0.0031
Over-speeding indicator (1 if an accident happened due to over-speeding, 0 otherwise) [SI]	1.163	8.32	−0.0029	−0.091	0.0953	−0.0015
Temporal Attributes						
Off-peak hour indicator (1 if an accident happened in off-peak hours, 0 otherwise) [FI]	−0.29	−2.61	0.0003	0.0075	0.0002	−0.0098
Weekday indicator (1 if an accident happened on a weekday, 0 otherwise) [FI]	0.418	3.59	−0.0007	−0.0167	−0.0043	0.0218
Number of observations	5067					
Number of parameters	19					
Log-likelihood at zero	−7024.353					
Log-likelihood at convergence	−3477.439					
ρ^2^ = 1 − *LL*(*β*)/*LL*(0)	0.504					

**Table 10:** Model estimation results for non-helmet-wearing motorcycle crashes 2018.

Variables	Parameter Estimates	*t*-Stats	Marginal Effects			
			No Injury	Minor Injury	Severe Injury	Fatal Injury
Constant [MI]	1.663	13.01				
Constant [SI]	1.981	13.5				
Constant [FI]	1.509	11.27				
Random parameter (normally distributed)						
Over-speeding indicator [MI]	5.085	3.64	−0.0044	0.0581	−0.0325	−0.0212
Heterogeneity in the mean of the random parameter						
Below-20 years indicator (1 if motorcyclist’s age lies below 20 years, 0 otherwise) [SI]	−1.184	−2.14				
Heterogeneity in the variance of the random parameter						
Large truck indicator (1 if a truck was involved in a crash, 0 otherwise) [FI]	0.527	2.28				
Roadway attributes						
Lane 2 indicator (1 if a crash occurred on 2 lane road, 0 otherwise) [NI]	−1.412	−2.71	−0.0010	0.0003	0.0005	0.0002
Rider Attributes						
Below-20 years indicator (1 if motorcyclist’s age lies below 20 years, 0 otherwise) [SI]	0.585	4.69	−0.0010	−0.0087	0.0153	−0.0056
Above 50 years indicator (1 if motorcyclist’s age is above 50 years, 0 otherwise) [FI]	1.538	10.23	−0.0006	−0.0102	−0.0085	0.0193
Crash Attributes						
Passenger car indicator (1 if an accident happened with a passenger car, 0 otherwise) [FI]	−3.824	−6.57	0.0002	0.0007	0.0011	−0.0019
Large truck indicator (1 if a large truck is involved in a crash, 0 otherwise) [FI]	−3.419	−3.25	0.0001	0.0001	0.0004	−0.0006
Violation Attributes						
Distraction indicator (1 if a crash occurred due to distraction, 0 otherwise) [NI]	−1.516	−5.11	−0.0038	0.0012	0.0014	0.0012
U-turn indicator (1 if crash occurred at U-turn, 0 otherwise) [FI]	−4.151	−4.14	0.0001	0.0003	0.0004	−0.0007
Temporal Attributes						
Weekday indicator (1 if an accident happened during non-weekend days, 0 otherwise) [SI]	0.399	4.24	0.002	0.016	−0.0293	0.0112
Number of observations	5566					
Number of parameters	15					
Log-likelihood at zero	−7716.114					
Log-likelihood at convergence	−4151.247					
ρ^2^ = 1 − *LL*(*β*)/*LL*(0)	0.458					

**Table 11:** Model estimation results for non-helmet-wearing motorcycle crashes 2019.

Variables	Parameter Estimates	*t*-Stats	Marginal Effects			
			No Injury	Minor Injury	Severe Injury	Fatal Injury
Constant [FI]	−0.542	−7.54				
Random parameter (normally distributed)						
Weekday indicator [SI]	−3.211	−5	−0.0034	−0.0396	0.0397	0.0033
Heterogeneity in the mean of the random parameter						
Over-speeding indicator (1 if a crash happened due to over-speeding, 0 otherwise) [MI]	2.096	10.82				
Heterogeneity in the variance of the random parameter						
U-turn indicator (1 if a crash occurred at U-turn, 0 otherwise) [FI]	0.314	2.08				
Roadway attributes						
Collector road indicator ( 1 if a crash occurred on collector road, 0 otherwise) [MI]	−0.23	−3.1	0.0027	−0.0082	0.0029	0.0026
Rider Attributes						
Below 20 years indicator (1 if motorcyclist’s age is below 20 years, 0 otherwise) [SI]	0.705	7.44	−0.002	−0.0131	0.0177	−0.0026
Above 50 years indicator (1 if motorcyclist’s age is above 50 years, 0 otherwise) [FI]	1.385	9.79	−0.001	−0.0124	−0.0049	0.0184
Crash Attributes						
Passenger car indicator (1 if an accident happened with a passenger car, 0 otherwise) [FI]	−2.404	−6.21	0.0003	0.0016	0.0003	−0.0022
Motorcycle indicator (1 if two motorcyclists collide with each other, 0 otherwise) [MI]	−0.639	−10.05	0.027	−0.087	0.0308	0.0292
Violation Attributes						
U-turn indicator (1 if crash occurred at U-turn, 0 otherwise) [FI]	−4.389	−4.39	0.0003	0.0002	0.0001	−0.0006
Over-speeding indicator (1 if a crash occurred due to over-speeding, 0 otherwise) [MI]	3.094	46.33	−0.1287	0.326	−0.1361	−0.0611
Distraction indicator (1 if a crash occurred due to distraction, 0 otherwise) [NI]	−4.041	−16.97	−0.0105	0.0039	0.0017	0.0049
Temporal Attributes						
Spring indicator (1 if a crash happened in the spring season, 0 otherwise ) [NI]	0.675	6.51	0.0101	−0.0081	−0.0014	−0.006
Number of observations	7202					
Number of parameters	14					
Log-likelihood at zero	−9984.092					
Log-likelihood at convergence	−5942.63					
ρ^2^ = 1 − *LL*(*β*)/*LL*(0)	0.405					

FI = fatal injury, SI = severe injury; MI = minor injury; NI = no injury.

## Data Availability

Data used in this research may be requested from the first author upon reasonable request.
